# Monitoring Cortical Excitability during Repetitive Transcranial Magnetic Stimulation in Children with ADHD: A Single-Blind, Sham-Controlled TMS-EEG Study

**DOI:** 10.1371/journal.pone.0050073

**Published:** 2012-11-21

**Authors:** Christian Helfrich, Simone S. Pierau, Christine M. Freitag, Jochen Roeper, Ulf Ziemann, Stephan Bender

**Affiliations:** 1 University Hospital for Child and Adolescent Psychiatry, Psychosomatics and Psychotherapy, Goethe University, Frankfurt/Main, Germany; 2 Section Clinical Neurophysiology and Multimodal Imaging, Child and Adolescent Psychiatric Hospital, Medical Faculty Carl-Gustav-Carus-University of Technology, Dresden, Germany; 3 Institute for Neurophysiology, Goethe University, Frankfurt/Main, Germany; 4 Department of Neurology and Stroke, Hertie-Institute for Clinical Brain Research, Eberhard-Karls-University, Tübingen, Germany; University of Montreal, Canada

## Abstract

**Background:**

Repetitive transcranial magnetic stimulation (rTMS) allows non-invasive stimulation of the human brain. However, no suitable marker has yet been established to monitor the immediate rTMS effects on cortical areas in children.

**Objective:**

TMS-evoked EEG potentials (TEPs) could present a well-suited marker for real-time monitoring. Monitoring is particularly important in children where only few data about rTMS effects and safety are currently available.

**Methods:**

In a single-blind sham-controlled study, twenty-five school-aged children with ADHD received subthreshold 1 Hz-rTMS to the primary motor cortex. The TMS-evoked N100 was measured by 64-channel-EEG pre, during and post rTMS, and compared to sham stimulation as an intraindividual control condition.

**Results:**

TMS-evoked N100 amplitude decreased during 1 Hz-rTMS and, at the group level, reached a stable plateau after approximately 500 pulses. N100 amplitude to supra-threshold single pulses post rTMS confirmed the amplitude reduction in comparison to the pre-rTMS level while sham stimulation had no influence. EEG source analysis indicated that the TMS-evoked N100 change reflected rTMS effects in the stimulated motor cortex. Amplitude changes in TMS-evoked N100 and MEPs (pre versus post 1 Hz-rTMS) correlated significantly, but this correlation was also found for pre versus post sham stimulation.

**Conclusion:**

The TMS-evoked N100 represents a promising candidate marker to monitor rTMS effects on cortical excitability in children with ADHD. TMS-evoked N100 can be employed to monitor real-time effects of TMS for subthreshold intensities. Though TMS-evoked N100 was a more sensitive parameter for rTMS-specific changes than MEPs in our sample, further studies are necessary to demonstrate whether clinical rTMS effects can be predicted from rTMS-induced changes in TMS-evoked N100 amplitude and to clarify the relationship between rTMS-induced changes in TMS-evoked N100 and MEP amplitudes. The TMS-evoked N100 amplitude reduction after 1 Hz-rTMS could either reflect a globally decreased cortical response to the TMS pulse or a specific decrease in inhibition.

## Introduction

Repetitive transcranial magnetic stimulation (rTMS) is a non-invasive tool used to induce long-term changes in cortical excitability by increasing or decreasing neuronal excitability and/or inhibition, depending on the chosen stimulation parameters [1]–[3] and the baseline level of cortical excitability [4].

Up to date, only few data exist on the immediate effects of rTMS on the cortex, and even less so about the developing cortex. A suitable imaging method is a mandatory safety issue in the process of making rTMS available for children with psychiatric disorders like ADHD as the neurophysiological and clinical effects of rTMS have not yet been established in children. A careful ‘online’ monitoring of rTMS effects in pediatric patients would be very helpful in the further process of establishing rTMS as a therapeutic tool [5].

In ADHD, hyperactivity is one of the cardinal clinical symptoms, with an imbalance between excitatory and inhibitory processes in the motor cortex contributing to its pathophysiology [6]–[8], expressed by reduction of short and long interval intracortical inhibition (SICI, LICI) [6], [7], [9]–[12] as well as intracortical facilitation (ICF) [6]. Compared to healthy controls children with ADHD showed a reduced TMS-evoked N100 [13] evaluated by single TMS pulses. Registered by multi-channel EEG, the N100 is a negative TMS-evoked potential (TEP) component with a latency of approximately 100 ms which decreased in amplitude during movement initiation and execution [14], [15] and increased during response inhibition [16]. Thus, the N100 likely reflects (motor) cortical inhibition [14]–[20].

Low frequency 1 Hz-rTMS (LFS) is of high therapeutic interest for ADHD, as it might normalize the excitation/inhibition balance in the motor system of patients with ADHD by increasing inhibition [5], [21]. LFS was able to reliably reduce motor cortex excitability as indexed by motor evoked potential (MEP) amplitudes [22]. After five days of 1 Hz-rTMS, one adult female ADHD patient showed reduced motor hyperactivity for four weeks [23]. So far, one study reported positive cognitive effects after high frequency rTMS of the right prefrontal cortex in adult ADHD patients [24]. Another pioneering study with healthy adults suggested that TEPs can be used to measure the effect of facilitatory high frequency rTMS on the primary motor cortex [25]. To date, no such data are available for the inhibitory 1 Hz-rTMS in children or adults.

This paper does not examine clinical changes in behavior after rTMS. Instead, it aimed at establishing how TEPs were influenced by subthreshold 1 Hz-rTMS and how these changes would correlate with changes in MEP amplitude, reflecting changes in motor corticospinal excitability. This investigation aimed to illustrate ‘online’ as well as short-term effects of 1 Hz-rTMS in children with ADHD in order to proof whether the N100 is a suitable ‘online’ marker of immediate rTMS effects on the cortex. To our knowledge this is the first study that monitors online rTMS effects in children with ADHD. The N100 was chosen as it is the most pronounced deflection of the TEP in children [14] and earlier components were difficult to separate from temporalis and facial muscle MEP artifacts. Previous studies reported that TEPs were not only induced by supra-threshold stimuli, but also by stimulation intensities below MEP threshold (resting motor threshold = RMT) [26], [27] like in the present study. The aim of the present study was to investigate whether the TMS-evoked N100 qualifies as a dynamic parameter that immediately reflects changes in cortical excitability throughout the course of rTMS.

## Materials and Methods

### 

#### 2.1.1. Ethics statement

Written informed consent was obtained from all parents and children prior to inclusion in the study which was approved by the local ethics committee (Goethe University of Frankfurt/Main) and conducted according to the Declaration of Helsinki [28]. The children received remuneration (50 Euro) following the study.

#### 2.1.2. Subjects

In our single-blind, sham-controlled study, twenty-five children with ADHD aged 8–14 years (mean age±standard deviation, 11.0±1.7 years, range 8.4–13.9; 23 male, 2 female) with an average intelligence quotient (CFT-20: mean±standard deviation, 96.6±12.2) participated (Table 1).

**Table 1:** Sample characteristics.

	Mean±Standard Deviation (SD)
**Age** (years)	11.0±1.7
**IQ**	96.6±12.2
**RMT** (50 µV) (N = 19) (% maximal stimulator output)	68%±11%
**80% of RMT** = stimulation intensity for 1 Hz-rTMS	54%±9%
**right-handedness (laterality index)**	83%±19%

(RMT = resting motor threshold; rTMS = repetitive transcranial magnetic stimulation).

Inclusion criteria followed actual safety guidelines [5]. We compared 1 Hz-rTMS to sham stimulation in a patient population who may benefit from a rTMS therapy-to-be-developed in the future. For ethical reasons we refrained from including healthy children as a control group (in addition to sham stimulation as a control condition) as there is no apparent direct benefit to be expected for these children. Apart from an N100 amplitude reduction, no qualitative differences in motor cortex TMS-evoked potentials have been found between children with ADHD and healthy children [13] when measured with 20 single TMS pulses.

The diagnosis of ADHD (DSM-IV 314.01) was verified by K-DIPS with the parents (Diagnostic Interview for Psychiatric Disorders in Children [29]) and direct clinical observation. Other neuropsychiatric disorders – like tics, depression, autism, etc. - were excluded. Neurological diseases like epilepsy, cerebral palsy, etc. were excluded likewise. Two children suffered from comorbid conduct disorder and three children had monosymptomatic nocturnal urinary incontinence (K-DIPS diagnoses). If a patient was currently being treated by medication with psychostimulants (n = 16; 64%), it was ceased for at least 48 hours prior to the study [30]. Right-handedness was assessed by the Edinburgh Handedness Inventory [31] and only right-handed children participated. Neurological soft signs were assessed by the Heidelberg neurological soft sign scale [32].

### 2.2. Transcranial Magnetic Stimulation

A MagPro X100 (MagVenture, Denmark) with a figure-of-eight-coil (outer diameter of each wing 75 mm) was used for this study. The magnetic pulses had a biphasic waveform. The coil was held over the dominant left motor cortex tangentially to the skull. The handle pointed postero-laterally with an angle of 45° to the midline of the subject’s head. The current flow over the left motor cortex was posterior-anterior. The coil was placed at the site that elicited maximum MEP amplitudes in the right first dorsal interosseous muscle (FDI). The coil weight was carried by a metal fastening arm. In addition, the experimenter manually assured that the position of head and coil remained constant during the stimulation and the recordings. A constant coil position throughout the recording session was assured by marks on the electrode cap indicating the point of optimal excitability for the right FDI. All children were sufficiently hearing protected.

### 2.3. Measurement of Cortical and Corticospinal Excitability via Single Pulse TMS

#### 2.3.1. Determining resting motor threshold

The resting motor threshold (RMT) of the relaxed right FDI was determined at the point of optimal excitability as the lowest stimulation intensity that produced MEPs with peak-to-peak amplitudes of ≥50 µV in at least 5 out of 10 consecutive trials [33] (Table 1).

If the maximum stimulator output (MSO) had reached 100% and no point of optimal excitability (POE) had been found yet (children have considerably higher motor thresholds than adults [13], [14]), further single pulses with 100% MSO were applied while the patient performed moderate muscle contraction to produce facilitation. Having located the POE of the FDI, later measurements and rTMS were both carried out at 100% MSO for these subjects (n = 6). As this study was especially interested in the time course of the TEP and focused on intraindividual rather than between-subject comparisons, these subjects were included on the basis that the subthreshold TMS is able to elicit N100 [26], [27], which was the case for all of these 6 subjects.

#### 2.3.2. Stimulation intensity (suprathreshold single pulse TMS before and after rTMS)

TEPs and MEPs to single TMS pulses at an intensity of “110% RMT” were measured before and immediately after 1 Hz-rTMS respectively sham-stimulation. When “110% RMT” exceeded the maximum stimulator output, the intensity was set at 100% MSO.

#### 2.3.3. Data acquisition and preprocessing (suprathreshold single pulse TMS before and after rTMS)

Twenty trials were recorded. Intertrial intervals varied randomly from 6 to 10 seconds to limit anticipation of the next trial. Only trials without artifacts were chosen for analysis. If a trial was rejected in the EEG, the corresponding trial in EMG was discarded as well, and vice versa [18]. The mean rejection rate was 1.4 trials (SD = 1.3; range = 0–5 trials). While for adults a greater number of trials is of advantage [34], children show higher N100 amplitudes than adults and due to the higher signal-to-noise-ratio [13], [14] a sufficiently large number of trials was analyzed [13], [14].

### 2.4. Modulation of Cortical Excitability by Low Frequency rTMS

#### 2.4.1. Rtms

All patients underwent a 1 Hz-rTMS protocol applied to the left primary motor cortex for fifteen minutes (900 stimuli in one continuous train; [5], [33], [35], [36]). The intensity for rTMS was set at 80% of the participant’s RMT. If no RMT was found at 100% MSO, rTMS were conducted at a stimulation intensity set at 100% MSO. This stimulation protocol is in accordance with current rTMS safety guidelines [5].

#### 2.4.2. Sham stimulation

Each participant also underwent sham stimulation either as the first or the second stimulation of the day (single-blind trial). The order of the two conditions was randomized and counterbalanced to control sequential effects. The delay between the two stimulation conditions amounted to 30 minutes. During the sham stimulation the deactivated coil was held over the skull, as like during rTMS, and coil clicks were presented via earphones for each stimulus (MagVenture sham stimulation). The subjects wore the earphones during both rTMS and sham stimulation.

### 2.5. Electroencephalographic Recordings

The registration of 64-channel DC-EEG was performed by a BrainAmps MR plus system (BrainProducts, Munich, Germany). EEG was recorded by equidistantly positioned sintered Ag-AgCl pin electrodes on elastic caps (FMS, Munich, Germany; extended international 10–20-system, minor deviations indicated by’). Electrode impedances were kept below 5 kOhm. Vertical and horizontal electrooculograms were registered by electrodes 1 cm above and below the left eye and next to the outer canthi. The EEG was digitized at an A/D-rate of 5 kHz. An anti-aliasing filter of 1000 Hz was applied. A high A/D rate together with wide filter settings was chosen in order to minimize TMS-artifacts. Fpz was chosen as a recording reference as this site experiences little influence from TMS induced artifacts due to its distance from the TMS site. The EEG was synchronized with TMS by TTL (transistor-transitor-logic)-triggers.

### 2.6. EEG Signal Preprocessing and Data Analysis

Evaluation of epileptiform EEG activity was conducted on the continuous recorded EEG data by an experienced EEG clinician (SB).

Subsequent data pre-processing was performed by the Vision Analyzer software (BrainProducts GmbH, Munich, Germany). Offline, data were re-referenced to the average reference. Segments 105 ms before to 900 ms after the TMS pulse were analyzed. The first 100 ms served as baseline. The baseline ended 5 ms before the TMS stimulus in order to avoid any contamination of the baseline by the TMS-artifact. As TMS artifacts render automatic artifact rejections impossible, in a first step, trials with strong non-TMS induced artifacts such as EMG activity or slow DC drifts were rejected by visual inspection. In a second step, in order to avoid negative influences of the TMS-induced artifact on further pre-processing steps such as eye-artifact correction [37], a shorter epoch was created after baseline correction (from 30 ms to 280 ms, both after TMS) in order to ‘cut the TMS-induced artifact out’. Following this, an ocular correction according to the algorithm described by Gratton & Coles was conducted (Brain Vision Analyzer).

The peak of the N100 was determined as the most negative potential during the interval of 80 ms to 140 ms in leads C3, CP3′ and CP5′. The average potential in a time-window of 40 ms centered on the detected peak (±20 ms) was calculated as TMS-evoked N100 amplitude. Leads C3, CP3′ and CP5′ were pooled for statistical analyses [14], [15]. In order to determine the TMS-evoked N100 before and after 1 Hz-rTMS/sham stimulation, averaged trials were compared (see 2.3.). Earlier components of the TEP may also be modulated by 1 Hz-rTMS [25], but they were not a target of this study due to their unclear maturational trajectories, and to difficulties separating very early components from TMS-elicited muscle twitches [38]. Thus, this study relied on N100, the most distinct TEP and reliable component, which is evoked by motor cortex stimulation in children [14].

### 2.7 rTMS Data Analysis

Continuous monitoring by TEP recording was performed during a 1 Hz-rTMS session of 900 stimuli. For statistical analysis, these were divided into nine trial blocks in steps of one hundred trials (1–100, 101–200, …, 801–900). Blocks were only formed to facilitate data analysis with a good signal-to-noise-ratio and because a direct regression analysis of all 900 single trial responses was technically difficult. We checked that no rapid changes especially during stimuli 1–100 were masked by this block-wise analysis (averages of first few trials) and that the reported results did not depend on the choice of the exact block size.

### 2.8 Electromyographic Recordings

Surface EMG (compound muscle action potential) of the right FDI was recorded in a belly-tendon montage (active electrode on the belly of the FDI, reference on the proximal phalanx of digit) by a bipolar amplifier BrainAmp ExG MR (BrainProducts, Munich, Germany) which was synchronized with the EEG recordings.

Motor evoked potentials (MEPs) were tested before and after 1 Hz-rTMS and sham stimulation for the 19 subjects, where a reliable RMT ≤100% MSO was found. Due to technical difficulties, no EMG recordings were available for one patient, and therefore 18 patients remained for the MEP analysis.

### 2.9 Source Analysis

In order to locate the cortical origin of the N100 respectively to verify its origin in the primary motor cortex, source analysis was performed. A PCA (principal component analysis) was conducted for the N100 time interval 80–140 ms for TEPs pre rTMS. The fully automated RAP-MUSIC algorithm (recursively applied and projected multiple signal classification; [39]) with subspace backprojection (SBSI = sequential brain source imaging; implemented in BESA research 5.3, BESA-GmbH, Munich, Germany) was applied to the group grand average pre rTMS. A singular topography was fitted on the two dimensional subspace with a minimum correlation of 90% (BESA default settings) because the first two principal components explained over 99% of the signal during the N100 time interval and the surface topography showed only a single pronounced left central peak. We checked that no qualitative change in the source structure occurred pre/post or during rTMS. As only the amplitude but not the source structure was changed, the source model was fitted to the TEPs pre rTMS and afterwards applied to the different stimulation blocks. This procedure is more adequate for localized activation, while global field power (GFP) [40]–[42] is usually applied for widespread phenomena, such as P300 [40], [41].

### 2.10 Statistics

Statistics were calculated using Statistica (StatSoft Inc., TX, USA).

The effects of 1 Hz-rTMS on the N100 amplitude were examined by a repeated measurements analysis of variance (rmANOVA).

1. Continuous monitoring during 1 Hz-rTMS:
  In a detailed analysis for the more subtle effects, nine trial blocks in steps of hundred stimuli (1–100, 101–200, …, 801–900) were examined in order to investigate the exact time course of rTMS influences on N100 amplitude.
  Linear and quadratic trends over the course of 1 Hz-rTMS were assessed. Additionally, Newman Keuls post hoc tests were calculated.
  In order to exclude influences on the order in which 1 Hz-rTMS and sham stimulation had been applied, a between-subjects-factor ORDER was also introduced into the rmANOVA. The factor ORDER should disentangle whether there were long-term effects of rTMS that would affect a later conducted sham stimulation.
2. N100 amplitudes in response to single TMS-stimuli:
  The preexistent, uninfluenced N100 (prior to 1 Hz-rTMS and sham stimulation) was compared to N100 amplitudes post 1 Hz-rTMS or sham stimulation. Therefore, a rmANOVA with the factor CONDITION (pre-stimulation versus after 1 Hz-rTMS versus after sham stimulation) was calculated. There was only one baseline condition at the beginning of the experiment, which was compared to 1 Hz-rTMS and the sham stimulation condition.
  The latter two conditions were conducted in a counterbalanced order; however a between-subjects-factor ORDER (1 Hz-rTMS before sham stimulation vs sham stimulation before 1 Hz-rTMS) was again introduced into the rmANOVA in order to disentangle whether 1 Hz-rTMS would have any longer-lasting effects which would influence the sham stimulation condition when performed post 1 Hz-rTMS.
  Greenhouse-Geisser correction was applied whenever the assumption of sphericity was violated. Statistical significance refers to a two-tailed p-value of <0.05.
3. MEP amplitudes: Correlations between the rTMS-induced amplitude changes of the N100 and of MEP amplitudes were determined using Pearson correlation coefficients. Additionally, differences in MEP amplitude and muscle precontraction levels were examined by a rmANOVA with the factor CONDITION (pre-stimulation versus after rTMS versus after sham stimulation) and the factor ORDER.

## Results

### 3.1. Clinical Assessment

No epileptic activity was observed in the EEG before, during or after 1 Hz-rTMS. RTMS was well tolerated, only three children reported mild transient headache.

### 3.2. TMS-evoked N100

#### 3.2.1 Monitoring TMS-evoked N100 during rTMS

A rmANOVA with nine blocks in steps of hundreds (1–100, 101–200, …, 801–900) showed a decrease in N100 amplitude (main effect trial block; F(8;192) = 3.7; Greenhouse-Geisser ε = 0.36; p = 0.02).

The N100 amplitude during blocks 3–9 differed from those during block 1 (Newman-Keuls post hoc tests; Table 2). In contrast, N100 amplitudes during blocks 5–9 did not differ from each other (Newman-Keuls post hoc tests: all p>0.75).

**Table 2:** Comparison of TMS-evoked N100 amplitudes between trial blocks throughout the 1 Hz-rTMS session (Newman Keuls post hoc tests).

	−15,9±24,5 µV	−13,5±23,5 µV	−11,7±23,2 µV	−10,6±21,5 µV	−10,0±18,8 µV	−8,6±19,5 µV	−8,9±21,3 µV	−9,5±21,4 µV	−10,3±22,9 µV
	1–100	101–200	201–300	301–400	401–500	501–600	601–700	701–800	801–900
1–100		0.18	**0.04**	**0.01**	**0.01**	**0.001**	**0.001**	**0.005**	**0.01**
101–200			0.28	0.20	0.26	0.08	0.10	0.18	0.25
201–300				0.54	0.79	0.57	0.62	0.72	0.72
301–400					0.95	0.86	0.88	0.92	0.88
401–500						0.83	0.80	0.75	0.87
501–600							0.84	0.86	0.85
601–700								0.75	0.85
701–800									0.88

Linear trend analysis revealed a significant overall N100 amplitude decrease (F(1;24) = 10.7; p = 0.003), which was still present when only the first half of trial blocks (1 to 5) were analyzed (F(1;24) = 6.6; p = 0.02), while there was no such trend for the second half of trial blocks (5 to 9; F(1;24) = 0.1; p = 0.81).

**Figure 1:**
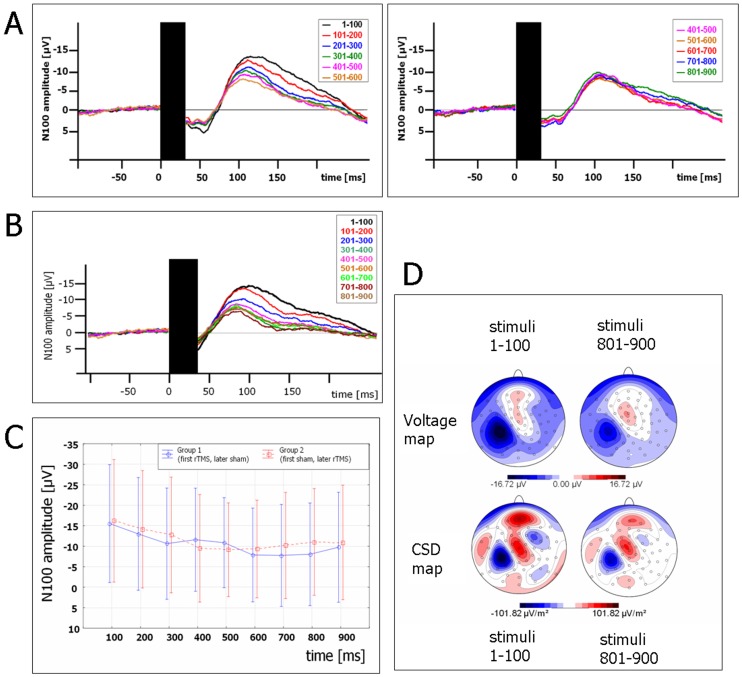
N100 amplitude decrease during 1 Hz-rTMS. (**A**) N100 amplitude reduction during 1 Hz-rTMS (group mean values). The TMS artifact (black box) has been cut out. Each curve represents an average of 100 trials (1–100, 101–200, …, 801–900). Electrodes C3, CP3’ and CP5’ were pooled. *Left*: TMS-evoked N100 amplitude continuously decreased during the stimuli 1–500. *Right*: N100 amplitude reached a plateau and was not further reduced by continued stimulation (pulses 500–900). (**B**) Single patient example. (**C**) TMS-evoked N100 amplitude was reduced during 1 Hz-rTMS regardless of the order of 1 Hz-rTMS vs. sham stimulation (blue: first 1 Hz-rTMS, second sham stimulation; red: first sham stimulation, second 1 Hz-rTMS; vertical bars show 0.95 confidence intervals). (**D**) Voltage and current source density (CSD) maps (blue areas indicate negativity, red areas positivity) show an N100 maximum above the stimulated left left central area and an intensity reduction during 1 Hz-rTMS. *Left*: N100 during stimuli 1–100. *Right*: N100 during stimuli 801–900.

Thus, the main N100 amplitude decrease occurred during the stimuli 1–500 (Figure 1 A/B), following which there was no further decrease in N100 amplitude but a stable plateau was observed.

#### 3.2.2 Comparison of rTMS and sham stimulation effects

Comparison of the N100 amplitudes to single TMS pulses pre and post 1 Hz-rTMS revealed a significant N100 amplitude reduction after 1 Hz-rTMS (main effect CONDITION F(2;48) = 3.4; p = 0.04; Greenhouse-Geisser ε = 0.97). Post hoc analysis (Newman Keuls post hoc tests) showed a significant difference between N100 amplitudes in pre- when compared to post- 1 Hz-rTMS (p = 0.035), while there was no difference between N100 amplitudes pre when compared to post sham stimulation (p = 0.86).

**Figure 2:**
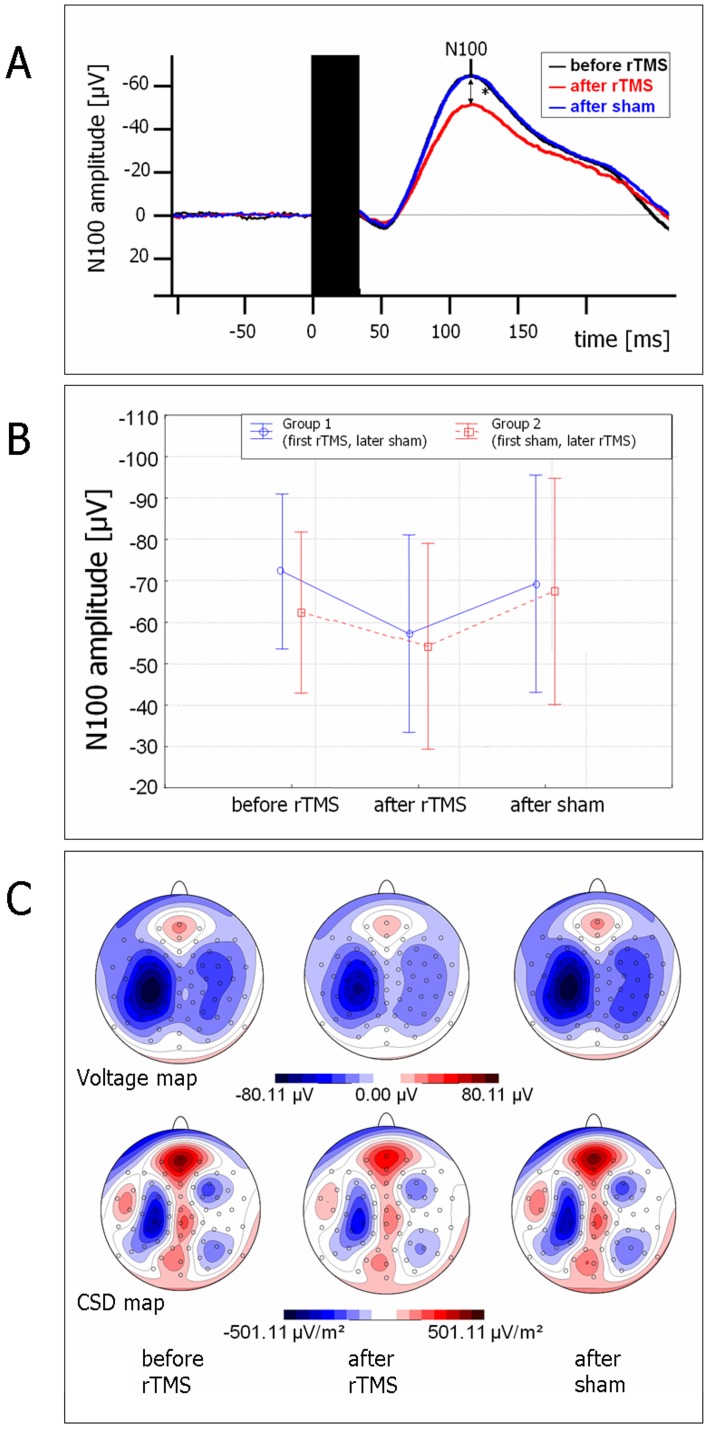
TMS-evoked N100 amplitude reduction after 1 Hz-rTMS. (**A**) Comparison of N100 before rTMS (black) with after 1 Hz-rTMS (red) and after sham stimulation (blue): N100 amplitude was only reduced after 1 Hz-rTMS. TMS artifact (black box) has been cut out. (**B**) N100 amplitude was reduced after 1 Hz-rTMS but not after sham stimulation irrespective of ORDER (blue: first 1 Hz-rTMS, second sham stimulation; red: first sham stimulation, second 1 Hz-rTMS; vertical bars show 0.95 confidence intervals). (**C**) Voltage and current source density (CSD) maps (blue for negativity, red for positivity) show TMS-evoked N100 localization above the stimulated left primary motor cortex and an intensity reduction after 1 Hz-rTMS.

Regardless of whether sham stimulation was performed before or following 1 Hz-rTMS, the N100 amplitude was reduced after 1 Hz-rTMS but not after sham stimulation (Figure 2): In the rmANOVA with the factors CONDITION (baseline, post 1 Hz-rTMS, post sham stimulation) and ORDER of 1 Hz-rTMS and sham stimulation, the influence of CONDITION remained significant (F(2;46) = 3.3; p = 0.047; Greenhouse-Geisser ε = 0.97), while there was no influence of the factor ORDER (F(1;23) = 0.1; p = 0.74). Moreover there was no interaction between the factors CONDITION and ORDER (p = 0.73) (Figure 2B).

#### 3.2.4 Source analysis

**Figure 3:**
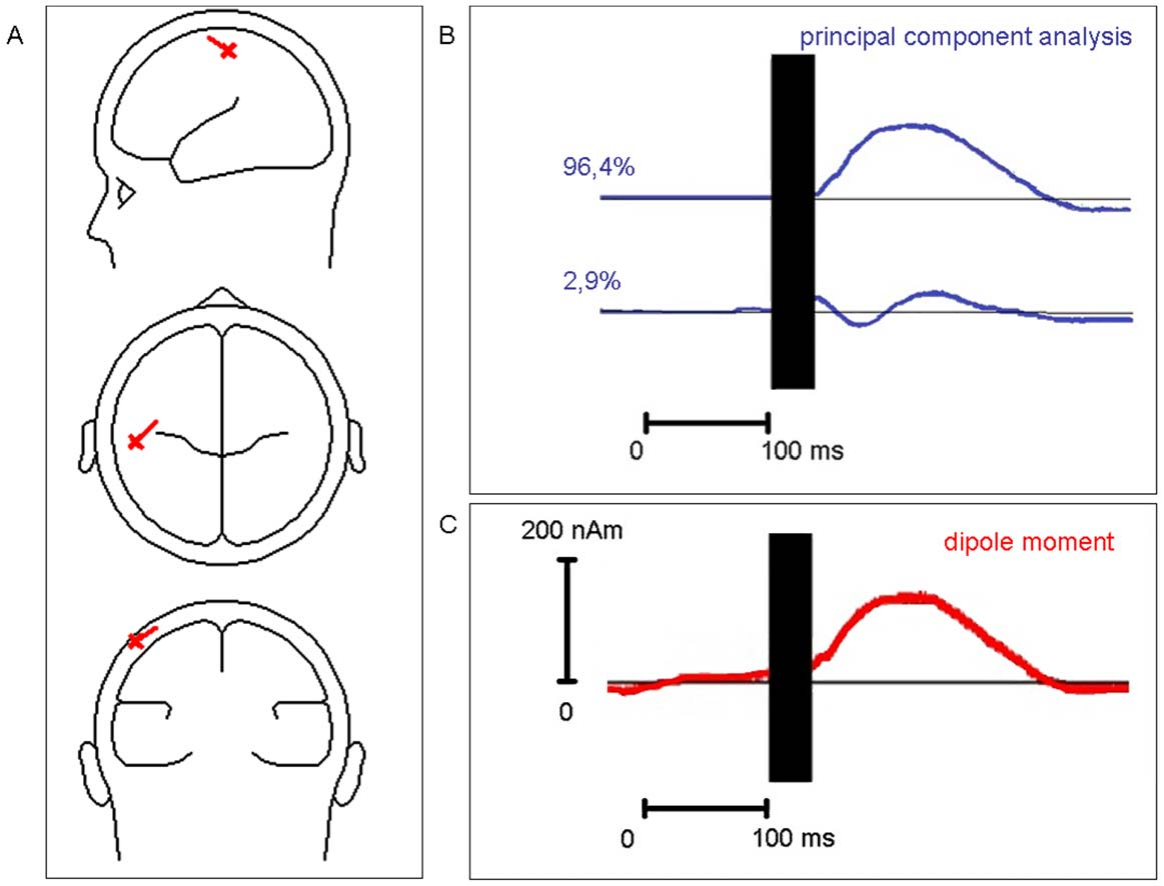
Source model of TMS-evoked N100. (**A**) The RAP-MUSIC (recursively applied and projected multiple signal classification) revealed a single source component located near the stimulated hand area of the primary motor cortex with an orientation approximately perpendicular to the precentral gyrus. (**B**) The first two principal components explained over 99% of the signal during the N100 time interval. The TMS artifact (black box) has been cut out. (**C**) The dipole moment of the single source component showed a maximum in the N100 interval. The TMS artifact (black box) has been cut out.

A single dipole component near the central sulcus explained the surface topography with a residual variance of 3.6% (time interval 80–140 ms) (Figure 3). Residual variance did not point towards further distinct N100 generators.

**Figure 4:**
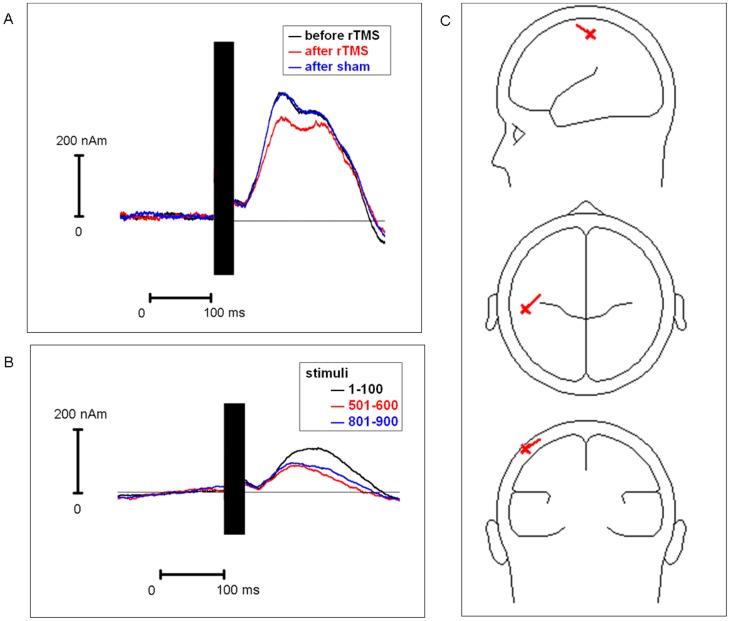
Dipole moment of N100 during, pre and post rTMS. (**A**) The momentum of the dipole component shown in the dipole model on the right (Figure 4C) is presented for the N100 time interval: before rTMS is shown in black, after 1 Hz-rTMS in red and after sham stimulation in blue. The TMS artifact (black box) has been cut out. (**B**) Momentum of the dipole component shown in the dipole model on the right (Figure 4C) during 1 Hz-rTMS. The TMS artifact (black box) has been cut out. The lines illustrate representative trial blocks at the beginning, in the middle and at the end of 1 Hz-rTMS (trials 1–100, 501–600 and 801–900). (**C**) Source model (cf. Figure 3).

For a graphical summary of rTMS influences on the momentum of the dipole component see Figure 4 and compare to Figures 1 and 2. Dipole moments correlated strongly with centro-parietal surface potentials at pooled leads C3, CP3′ and CP5′ (explained variance R^2^ = 97%; t = 28.0; p<0.0001 for the rTMS stimuli 1–100), thus statistics with individual dipole moments confirmed all surface potential results given above (no details presented in order to avoid redundancies). There were no qualitative differences between the source model for stimuli 801–900 compared to the source model for stimuli 1–100.

### 3.3. Correlation between rTMS-effects on TMS-evoked N100 and on MEP Amplitudes

**Figure 5:**
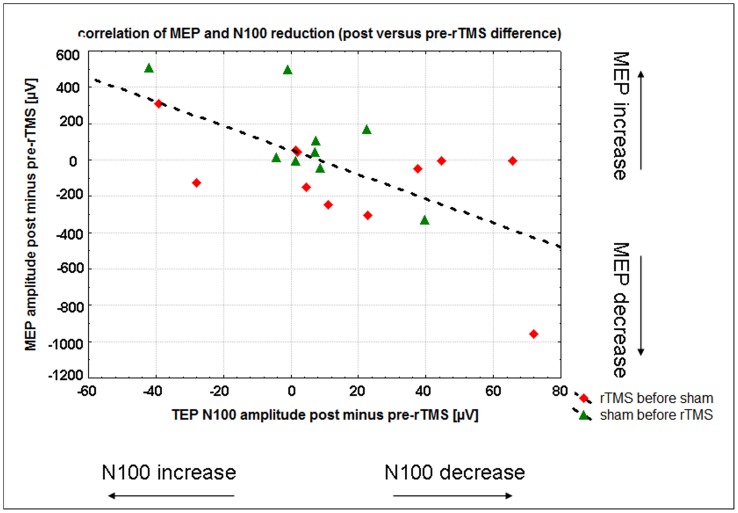
Correlation between amplitude changes of TMS-evoked N100 and MEP. The correlation between the rTMS induced change of N100 amplitude and the rTMS induced change of MEP amplitude is illustrated. The calculated difference ‘post-rTMS - pre-rTMS’ for the MEP means that negative values indicate a MEP amplitude reduction, and a positive value indicates an increase in MEP amplitude. Note that the same calculation for the TMS-evoked N100 amplitude (being a negative value) indicates a N100 amplitude increase (more negative N100) if the ‘post-rTMS - pre-rTMS’ difference is negative. Therefore, a less negative TMS-evoked N100 is accompanied by a lower MEP amplitude after 1 Hz rTMS.

MEP amplitudes pre-rTMS (185±257 µV), post-1 Hz-rTMS (164±203 µV) and post-sham stimulation (167±148 µV) did not differ (F(2;32) = 0.02; p = 0.95; GG-epsilon = 0.78). Pre-contraction levels (root mean square of the EMG for the time interval 500 ms to 5 ms pre TMS) also did not differ between the conditions (F(2;32) = 1.1; p = 0.34; GG-epsilon = 0.92: pre-rTMS 343±286 µV; post rTMS 397±300 µV; post sham stimulation 481±324 µV). Changes in N100 amplitude after 1 Hz-rTMS (comparison pre rTMS vs. post 1 Hz-rTMS) correlated significantly with changes in MEP amplitudes (N = 18; r = −0.66; t = 3.6; p = 0.003; Figure 5). I.e. reduced N100 amplitudes (less negative) were associated with reduced MEP amplitudes (less positive). However, the correlation between MEP and N100 amplitude changes was found also between the pre-rTMS and post sham stimulation conditions (N = 18; r = −0.59; t = 2.9; p = 0.01), though the association was descriptively weaker in this condition.

There was no significant correlation between N100 amplitude reduction and age (r = 0.01; p = 0.95) nor between N100 amplitude reduction and absolute rTMS intensity (r = −0.26; p = 0.21).

## Discussion

The TMS-evoked N100 decreased throughout the 1 Hz-rTMS session. Thereby, the N100 amplitude decrease during the first half was more pronounced than during the second half of the 900 rTMS pulses, suggesting that the effect of rTMS protocols on cortical excitability may saturate when a certain number of pulses is exceeded. When N100 amplitudes were measured by suprathreshold single-pulse TMS before and after 1 Hz-rTMS, the N100 reduction was confirmed. The N100 reduction only occurred after 1 Hz-rTMS but not after sham stimulation. The N100 data obtained in this study illustrate that 1 Hz-rTMS modulated cortical excitability for at least 10 minutes after the end of the rTMS in ADHD children (time between rTMS and the assessment of the EEG responses to single pulse TMS). These are the first of such data about immediate cortical rTMS effects in children. The duration of rTMS effects can only be estimated and was clearly shorter than 45 minutes as measurements after sham stimulation (in the group with prior 1 Hz-rTMS) showed no persisting effects. Thus, the factor ORDER of rTMS and sham stimulation had no effect.

In our sample the MEP amplitude showed a non-significant descriptive reduction after both 1 Hz-rTMS and sham stimulation; although most (but not all) previous studies reported a significant MEP reduction after 1 Hz-rTMS [21], [22], [33]. Maybe statistical power was not high enough to detect an MEP reduction in our study or TMS intensity was too weak to exert significant effects on the MEP. RTMS-related changes in N100 could be related to the same processes as rTMS-related MEP changes. However, in our sample, TMS-evoked N100 amplitude turned out to be a more sensitive marker for rTMS-induced changes than MEP amplitude.

The TMS-evoked N100 amplitude qualified as a successful ‘online’ marker for monitoring changes in cortical excitability during 1 Hz-rTMS in ADHD children. Thus, this study contributes to establishing TEPs as a useful approach for such an objective monitoring, which is described here for the first time in a neuropsychiatric pediatric patient group. As the TMS-evoked N100 has been successfully elicited in healthy children and adults as well [13], [23], [24], TEP monitoring of rTMS effects might also be employed in those groups and may not be limited to children with ADHD. The hypothesis that 1 Hz-rTMS changes the N100 amplitude in a similar way also in healthy children or adults seems plausible but remains yet to be proven in future studies. In any case, we would like to point out clearly that the current findings may not be specific for or limited to children with ADHD.

Other pioneering pilot studies are pointing towards a possible therapeutic application of rTMS in ADHD (1 Hz inhibitory primary motor cortex rTMS [23]; excitatory high frequency rTMS to the DLPFC [24]). Our approach to establish non-invasive online monitoring parameters of short-term effects of rTMS on the cortex may in the long run help to optimize rTMS protocols: If the TMS-evoked N100 reduction (to be more precise: a less negative potential) indicated a decrease in inhibition – as previous studies reported reduced N100 amplitudes in experimental conditions of decreased cortical inhibition [14], [16], [34] –, it would have to be concluded that 1 Hz-rTMS reduced inhibition instead of strengthening it. If that was the case, low frequency 1 Hz-rTMS might not be the most appropriate therapeutic approach in ADHD. Instead, another stimulation protocol may be more useful for ADHD children. This line of interpretation assumes that 1 Hz-rTMS directly influenced the TMS-evoked N100 amplitude and thereby reduced cortical inhibition. It has been shown that 1 Hz-rTMS can decrease both excitatory and inhibitory processes in the cortex [43]–[45].

Nevertheless, 1 Hz-rTMS might not have influenced the N100 amplitude directly, but rather altered the level of cortical excitability. Therefore, the cortical inhibition might be increased, e.g. due to membrane potential shifts, and subsequently a TMS pulse of the same intensity would lead to less excitation. Then the N100 amplitude reduction would be a result of a weaker cortical excitation by the TMS stimulus and would rather reflect reduced cortical excitability than decreased inhibition. Although a descriptively very slight decrease of MEP amplitudes would support this hypothesis, this reduction occurred after both 1 Hz-rTMS and sham stimulation. Further studies are needed to resolve the exact neurophysiological mechanisms behind the rTMS induced changes in TMS-evoked N100 amplitude.

Considering that changes (“after minus before”) in TMS-evoked N100 (as a putative measure of an inhibitory brain response to limit the TMS-related excitation [13], [14], [34]) and MEP amplitude (as a measure of motor cortex excitability) correlated significantly for both 1 Hz-rTMS and for sham stimulation, this could indicate that 1 Hz-rTMS was able to affect inhibitory and excitatory systems in the same direction at the same time; although with varying effectiveness, maybe due to different independent thresholds. A parallel reduction in MEP amplitude as a measure of cortical excitability and measures of motor cortical inhibition (e.g. SICI) has also been reported in other studies [43]–[45].

However, considering the data from our sample, no definite decision can be made whether TMS-evoked N100 and MEP modulations are mechanistically independent of each other or linked. Being linked TMS-evoked N100 changes could be a direct consequence of the same changes reflected by MEP amplitude. In this case, the decreased N100 after 1 Hz-rTMS would reflect that the post-rTMS excitation was relatively weaker (as reflected by MEP decreases after 1 Hz-rTMS in other studies), and – as a result – the reactive inhibition during N100 was less pronounced as well. As, on the other hand, MEP amplitudes were comparable after 1 Hz-rTMS and sham stimulation, this interpretation may be less likely. Correlations between TMS-evoked N100 and MEP changes were found also in the sham condition and could rather reflect changes in vigilance than rTMS-specific effects.

If rTMS effects on MEP and TEP were independent, as MEP and TMS-evoked N100 have been found to dissociate in several previous studies [13], [14], [34], the TMS-evoked N100 could yield additional information apart from the MEP. However, the dissociations in previous studies were not related to rTMS effects. Because there *was* a correlation of TMS-evoked N100 and MEP amplitudes pre versus post 1 Hz-rTMS, further studies are needed to clarify in how far the rTMS-related modulation of TMS-evoked N100 and MEP amplitudes are related to each other.

Additionally, this study does not provide any evidence that 1 Hz-rTMS leads to epileptiform activity in the EEG in children with ADHD. Similar to previous studies, 1 Hz-rTMS was well tolerated [46], [47]. No severe adverse events were observed, particularly no seizure or syncope, as was also indicated in previous TMS studies in children [5], [9], [33], [47]. For investigating new rTMS protocols and optimizing established ones it is imperative to maintain a distinct supervision of safety aspects, especially in children. Therefore, under careful monitoring, further data on rTMS in children should be acquired via controlled and monitored studies, in order to gain sufficient data to recommend specific rTMS protocols as safe for children. Together with clinical EEG, TEP monitoring can serve to assess the immediate online effects of rTMS on cortical excitability (N100 amplitude changed during 1 Hz stimulation) and thus also may serve as a safety measure. As the N100 reflects inhibitory processes, rapid changes in TMS-evoked N100 amplitude may provide additional information on the risk that a rTMS protocol might trigger epileptiform activity. For example, a strong N100 increase could indicate an increase in cortical excitability, as stronger TMS pulses lead to higher amplitudes in all TMS-evoked potential components, both excitatory as well as inhibitory. However, such speculations need to be tested in further studies. Under EEG and TEP monitoring, unexpected strong increases in cortical excitability can even be detected during subthreshold rTMS of the primary motor cortex. Therefore, the data presented in this study suggest that the N100 amplitude may be useful as an indicator to maximize the functional effects of rTMS on the cortex. As TMS-evoked N100 has also been described after prefrontal cortex stimulation [26], this TEP-monitoring could also be extended for surveying non-motor target areas [48] in further studies, even though different cortical areas may vary in their susceptibility and reactivity to rTMS [26], [27], [49]. Changes in TMS-evoked N100 amplitude could indicate if a certain rTMS protocol exerts effects on the (non-motor) cortex and their intensity could be estimated. TMS parameters could be varied to maximize rTMS effects on TMS-evoked potentials. During online monitoring, the TMS-evoked N100 amplitude provides immediate information about rTMS effects on the cortex even before conducting further tests.

### Technical Considerations

The topography of the N100 (Figures 1C, 2C) revealed a lateralized, negative maximum over the left central area overlying the stimulated motor cortex. This topography was not different when compared before and after rTMS. The central negativity is compatible with activation of the stimulated motor cortex despite a TMS intensity below the motor threshold.

Clearly, the N100 does not represent an auditory response elicited by the TMS click [14], [15] as auditory evoked potentials have smaller amplitudes and are less lateralized. Source analysis showed that the equivalent dipole component was located near the motor hand area (Figures 3A, 4C). Therefore, volume conduction from the temporal auditory cortex could be excluded as a major source of the centro-parietal N100. There was no deep temporal positivity which would have to be the case for potentials in the auditory cortex [50]–[52]. That rTMS effects on N100 persisted for some time after 1 Hz-rTMS and occurred only after 1 Hz-rTMS but not auditory sham stimulation is also incompatible with the notion that the reduction in N100 amplitude was caused by auditory habituation.

Muscle tension is unlikely to account for a decrease in N100 amplitude. Increased muscle contraction towards the end of the 1 Hz-rTMS could have led to an N100 decrease [15], however, this would have presented in combination with increased MEP amplitudes. In addition, quantitative EMG analysis did not show significant changes in muscle pre-contraction throughout the experiment and no muscle contraction differences between 1 Hz-TMS and sham stimulation.

The decrease in N100 amplitude occurred during rTMS at subthreshold intensity. Thus, no re-afferent tactile or proprioceptive evoked potentials were elicited [53]–[55], which could have been influenced indirectly by reduced MEP amplitudes after rTMS.

Further studies will have to provide evidence of a correlation between TEP changes and alterations in more complex behavioral parameters or clinical symptoms. Stimulation time and number of stimuli/sessions might have been too low to exert optimal clinically relevant behavioral effects.

### Conclusions

The present study investigated the online effects of subthreshold 1 Hz-rTMS on electrophysiological parameters of cortical excitability and illustrates, for the first time, that 1 Hz-rTMS yields (short-term) effects on the N100 amplitude in children with ADHD that may be used to monitor rTMS effects on cortical excitability. This might be relevant not only for therapeutic approaches to ADHD but also for other diseases. Future studies will have to assess whether the TMS-evoked N100 amplitude reduction after 1 Hz-rTMS reflects a globally decreased cortical response to the TMS pulse or a specific decrease in inhibition. In the long term, it will be necessary to assess summation effects after repeated treatments with 1 Hz-rTMS and the relevance of changes in TEPs to behavioral or cognitive processes. The N100 as marker of rTMS online monitoring may contribute to assuring safety of rTMS applied in experimental protocols or when used for investigations in risk groups or new patient populations like children or adolescents. RTMS may prove to be useful in various conditions of child and adolescent psychiatry, however, the specific aspects of the developing brain must be carefully investigated.
